# Cross-sectional survey of the undergraduate rheumatology curriculum in European medical schools: a EULAR School of Rheumatology initiative

**DOI:** 10.1136/rmdopen-2018-000743

**Published:** 2018-09-21

**Authors:** Abhishek Abhishek, Annamaria Iagnocco, J W J Bijlsma, Michael Doherty, Frédéric Lioté

**Affiliations:** 1 Academic Rheumatology, Faculty of Medicine and Health Sciences, School of Medicine, University of Nottingham, Nottingham, UK; 2 Dipartimento di Scienze Cliniche e Biologiche, Università degli Studi di Torino, Turin, Italy; 3 Department of Rheumatology and Clinical Immunology, University Medical Center Utrecht, Utrecht, The Netherlands; 4 Université Paris Diderot, USPC, UFR de Médecine, Paris, France; 5 Service de Rhumatologie (centre Viggo Petersen), Hôpital Lariboisière (AP-HP), Paris, France

**Keywords:** multidisciplinary team-care, treatment, disease activity

## Abstract

**Objectives:**

To survey the undergraduate rheumatic and musculoskeletal diseases (RMDs) curriculum content in a sample of medical schools across Europe.

**Methods:**

The undergraduate musculoskeletal diseases and disability curriculum of University of Nottingham, UK, was used as a template to develop a questionnaire on curriculum content. The questionnaire elicited binary (yes/no) responses and included the option to provide additional information as free text. The survey was mailed to members of the European League Against Rheumatism (EULAR) School of Rheumatology (Undergraduate Classroom) and to EULAR Standing Committee on Education and Training members in January 2017, with a reminder in February 2017.

**Results:**

Responses were received from 21 schools belonging to 11 countries. Assessment of gait, hyperalgesic tender site response and hypermobility were not included in many curricula. Similarly, interpretation of investigations undertaken on synovial fluid was taught in only 16 schools. While disease-modifying anti-rheumatic drugs and biological agents, and urate-lowering treatment were included in the curricula of 20 and 21 institutions, respectively, only curricula from 18 schools included core non-pharmacological interventions. Osteoarthritis, gout, rheumatoid arthritis, spondyloarthropathy, polymyalgia rheumatica and lupus were included in the curriculum of all institutions. However, common RMDs such as calcium pyrophosphate deposition, fibromyalgia, giant cell arteritis and bone and joint infection were included in 19 curricula.

**Conclusion:**

This survey highlights areas of similarities and differences in undergraduate curricula across Europe. It is hoped that the results of this survey will catalyse the development and agreement of a minimum core European Curriculum for undergraduate education in RMDs.

**Key messages**

**What is already known about this subject?**

- Surveys of medical undergraduate rheumatic and musculoskeletal disease (RMD) curricula in individual European countries suggest variations in curriculum content.
- A survey of undergraduate RMD curriculum in medical schools across Europe has not been performed.

**What does this study add?**

- This survey highlights areas of similarities and differences in undergraduate curricula across Europe.

**How might this impact on clinical practice?**

- It is hoped that the results of this survey will catalyse the development and agreement of a minimum core European Curriculum for undergraduate medical education in RMDs.

## Introduction

The European League Against Rheumatism (EULAR) Standing Committee on Education and Training (ESCET) published guidelines for rheumatic and musculoskeletal diseases (RMDs) undergraduate core curriculum in 1999, and there have been other attempts to harmonise the curriculum of undergraduate teaching in RMDs.^1 2^ Since then, some countries have developed curricula for RMDs, for example, France. However, surveys in individual European countries, the USA and the Asia-Pacific region demonstrated substantial variations in curriculum content.^3–6^ The content of undergraduate curricula for RMDs in medical schools across Europe has not been compared. This is necessary to provide a framework for developing pan-European educational resources and to encourage harmonisation of curricula in RMDs. With this in mind, the objective of this study approved by the EULAR School of Rheumatology (ESoR) (Undergraduate Classroom) was to survey the undergraduate RMD curricula in European medical schools.

## Methods

The undergraduate RMDs curriculum of University of Nottingham is based on the ESCET guidance^1^ and was used to develop a questionnaire. The survey document elicited yes/no responses and included the option for providing additional detailed information. This was reviewed by ESoR (Undergraduate Classroom) to assess face validity and was emailed to members of the ESoR (Undergraduate Classroom) and ESCET, in January 2017, with reminder in 4 weeks.

## Results

Twenty-one schools from 11 countries responded (online supplementary table S1). The questionnaires were completed by tenured faculty with teaching responsibilities and knowledge of their curriculum.

10.1136/rmdopen-2018-000743.supp1 Supplementary data

All schools taught differentiation between inflammatory and mechanical joint pain, and to make observations about the musculoskeletal system (online supplementary table S2). The identification and characterisation of symptoms and signs of arthropathy at hand, wrist, elbow, gleno-humeral, hip, knee, ankle and foot joints was taught in >90% of the responding institutions. In contrast, identification of periarticular lesions was taught in five institutions. However, students were expected to be aware of these conditions in majority of institutions (online supplementary figure S1). All institutions gave students opportunity to examine patients. Over 95% of the responding institutions taught the differential diagnosis of acute and chronic monarthritis, oligo-arthritis and inflammatory polyarthritis.

Investigations relevant to RMDs and general principles of management were included in most curricula (table 1, online supplementary figure S2, table S3). Common RMDs such as osteoarthritis, neck and low back pain, fibromyalgia and regional pain, bone diseases and crystal deposition diseases were included in majority of curricula with some variation in content (online supplementary figures S3–5, table S4). There was uniformity in coverage of autoimmune rheumatic diseases (AIRDs) in most curricula (table 2). While rare manifestations such as atlanto-axial subluxation in rheumatoid arthritis were included in 17 curricula, an exploration of long-term physical, psychological and social effects or the contribution of the multi-disciplinary team (MDT), and drug counselling and monitoring were only included in one curriculum. An outline of appropriate management plan for comorbidities was included in only two curricula.

**Table 1:** Curriculum content for management of common and uncommon RMD conditions

Condition	N (% schools)
Osteoarthritis	20 (95.2%)
Chronic back pain	18 (85.7%)
Nerve root entrapment	19 (90.5%)
Fibromyalgia	17 (81%)
Osteoporosis including primary preventions	20 (95.2%)
Gout including treatment to target	20 (95.2%)
CPP deposition disease	16 (76.2%)
Apatite crystal deposition	13 (61.9%)
Rheumatoid arthritis	20 (95.2%)
Spondyloarthritis	20 (95.2%)
SLE	21 (100%)
Anti-phospholipid syndrome	19 (90.5%)
Systemic sclerosis	19 (90.5%)
Sjögren’s syndrome*	18 (85.7%)
Polymyalgia rheumatica	21 (100%)
Giant cell arteritis	19 (90.50%)
Systemic vasculitis	18 (85.7%)
Inflammatory myopathies	17 (81%)
Bone/joint infection	19 (90.5%)
Osteomalacia	16 (76.2%)
Osteonecrosis	15 (71.4%)
Paget’s disease of bone	15 (71.4%)

*Either primary or secondary.

CPP, calcium pyrophosphate; RMD, rheumatic and musculoskeletal disease; SLE, systemic lupus erythematosus.

**Table 2:** Curriculum content for autoimmune inflammatory rheumatic diseases

	RA	SpA spectrum	SLE (APS)	Systemic sclerosis	Sjögren’s syndrome	Inflammatory myopathies	Systemic vasculitis	Giant cell arteritis	Polymyalgia rheumatica
Pathology	21	20	21	19	19	–	–	19	–
Clinical feature	21	20	21 (20)	20	19	18	20	19	21
Outcome	21	20	21	19	19	–	–	19	–
Investigations	21	20	21 (20)	20	20	17	19	19	21

APS, anti-phospholipid syndrome; RA, rheumatoid arthritis; SLE, systemic lupus erythematosus; SpA, spondyloarthritis.

The risk factors, common causative organisms, signs and symptoms, differential diagnosis, and investigation of bone and joint infection were included in 19 curricula. Bony malignancy including metastases were included in fewer curricula (online supplementary table S6), while uncommon conditions were included in very few institutions (online supplementary table S7).

## Discussion

This is the first survey of undergraduate RMDs curriculum of a number of medical schools across several European countries. It found areas of harmony and differences in curriculum content. There were similarities in teaching on AIRDs, while discrepancies were obvious on assessments such as gait examination, periarticular assessment and assessment for hypermobility. Similarly, identification of disability and role of MDT were taught in few institutions. Some medical schools included advanced imaging techniques with relevance to RMDs, while over 20% schools did not teach investigations undertaken on synovial fluid. While pharmacological management of autoimmune and other rheumatic diseases was included, there was a lack of teaching on adjunctive therapies and coping strategies. Factors underpinning variation in curriculum content may include different roles of rheumatologists across Europe and the need to tailor training to match local demands. The role of a rheumatologist depends on the number of rheumatologists per capita, postgraduate training and competing management of RMDs by other specialists such as orthopaedics, physiotherapists and general practitioners. Given the small number of medical schools that responded, we are unable to provide geographic comparisons.

Currently, a nationwide curriculum exists in France, but not in many other European countries to the best of our knowledge. It is hoped that the results of this survey will catalyse the development of a core European curriculum for undergraduate medical education in RMDs. The aim of undergraduate medical education has moved from acquisition of knowledge to acquiring competence, and this may make it easier to harmonise curricula across Europe once the expected competencies are standardised. Development of a core curriculum is likely to improve the amount of time devoted to teaching about RMDs. For instance, a recent survey of Canadian medical schools observed that an average of just 2.3% of their curriculum time was devoted to education about RMDs, despite these disorders being responsible for 13.7%–27.8% of all primary care consultations in Canada.^7^ Additionally, it will also improve the coverage of musculoskeletal topics in undergraduate textbooks.^8^

Research suggests that even when a condition is included in the curriculum, the delivery of teaching may be variable, and recently qualified doctors may have substantial deficits in their knowledge base as exemplified in a study in which majority of recently qualified doctors did not demonstrate competence about long-term management of gout.^9^

There are several caveats to our findings. First, this is a small sample of European medical schools, so generalisability may be limited. Second, we have not collected data on the amount of teaching, the teaching methods used, the detail to which each topic is taught, specific learning objectives and the quantity of exposure to patients. We feel that these data would be difficult to collect reliably as medical schools have several associated hospitals where the delivery of teaching occurs, and this may vary from place to place.

In conclusion, this survey highlights areas of similarities and differences in undergraduate curricula across Europe. It is hoped that it will catalyse the development of a core European Curriculum for undergraduate education in RMDs.

## References

[1] Doherty M, Woolf A Guidelines for rheumatology undergraduate core curriculum. Ann Rheum Dis 1999;58:133–5. 10.1136/ard.58.3.133 10364908 PMC1752843

[2] Woolf AD, Walsh NE, Akesson K Global core recommendations for a musculoskeletal undergraduate curriculum. Ann Rheum Dis 2004;63:517–24. 10.1136/ard.2003.016071 15082481 PMC1754992

[3] McColl G A survey of the musculoskeletal curricula of medical schools in the Asia-Pacific region. APLAR J Rheumatol 2005;8:84–9. 10.1111/j.1479-8077.2005.00138.x

[4] Bernstein J, Garcia GH, Guevara JL, et al Progress report: the prevalence of required medical school instruction in musculoskeletal medicine at decade’s end. Clin Orthop Relat Res 2011;469:895–7. 10.1007/s11999-010-1477-3 20683689 PMC3032845

[5] Kay LJ, Deighton CM, Walker DJ, et al Undergraduate rheumatology teaching in the UK: a survey of current practice and changes since 1990. Rheumatology 2000;39:800–3. 10.1093/rheumatology/39.7.800 10908701

[6] Riemekasten G, Aringer M, Baerwald CG, et al [Rheumatology—Integration into student training (RISA): current structure of clinical rheumatology in German universities (RISA III)]. Z Rheumatol 2016;75:493–501. 10.1007/s00393-016-0079-1 27193335

[7] Pinney SJ, Regan WD Educating medical students about musculoskeletal problems. Are community needs reflected in the curricula of Canadian medical schools? J Bone Joint Surg Am 2001;83-A:1317–20. 11568192

[8] Kay LJ, Coady DA, Walker DJ Joints: if relevant. Do available textbooks contain adequate information about musculoskeletal examination skills for medical students? Med Teach 2001;23:585–90. 10.1080/014215901200901032 12098480

[9] Terrill M, Riordan J A survey on the beliefs and knowledge of gout management in new medical graduates—New South Wales, Australia. Int J Rheum Dis 2018;21:517–22. 10.1111/1756-185X.13097 28544467

