# Supplementary material for: Cross-sectional survey of the undergraduate rheumatology curriculum in European medical schools: a EULAR School of Rheumatology initiative
Source: RMD Open. 2018 Sep 21;4(2):e000743. doi: 10.1136/rmdopen-2018-000743 (PMC6157535; doi:10.1136/rmdopen-2018-000743)
Supplement: Supplementary data [file rmdopen-2018-000743supp001.docx]

Table S1: Responding countries and the number of medical schools from each country

| Country | Number of schools |
| --- | --- |
| Spain | 4 |
| Italy | 4 |
| Germany | 3 |
| United Kingdom | 3 |
| Portugal | 1 |
| Switzerland | 1 |
| France | 1 |
| The Netherlands | 1 |
| Hungary | 1 |
| Poland | 1 |
| Czech Republic | 1 |

Table S2: Teaching on RMDs history and examination

| Topic | Number of schools |
| --- | --- |
| Inspection, palpation, and examination of movements | 18 |
| Gait-arm-leg-screen (GALS) | 14 |
| Description of the main phases of gait | 8 |
| Identification of specific gaits |  |
| Spastic | 13 |
| Antalgic | 12 |
| Trendelenburg | 11 |
| Parkinsonian | 10 |
| Identification of disability and participation restriction | 11 |
| Hyperalgesic tender sites for fibromyalgia | 16 |
| Screening assessment for hypermobility (Beighton’s score) | 7 |
| Cutaneous involvement in systemic sclerosis | 20 |

Table S3: Principles of management of RMDs

| Topic | Number of schools |
| --- | --- |
| Core non-pharmacological interventions |  |
| Heat/cold packs | 17 |
| Splinting | 14 |
| TENS | 10 |
| Exercise | 17 |
| Lifestyle interventions | 18 |
| Weight loss | 19 |
| Factors influencing adherence to management | 13 |
| Outline of coping strategies | 13 |
| Pharmacologic interventions |  |
| Analgesics | 18 |
| World Health Organisation pain ladder | 9 |
| Outline of the indication for epidural analgesia | 6 |
| Method of action, indications, contra-indications, side-effects: |  |
| Glucocorticoids | 18 |
| DMARDs and biological agents | 20 |
| Urate lowering treatment | 21 |
| Neuro-pharmaceuticals e.g. low dose amitriptyline | 1 |
| Nutraceuticals and alternative medicines | 8 |
| Surgical |  |
| Total joint replacement | 17 |
| Soft tissue surgery | 14 |
| Tendon transfers | 6 |
| Osteotomy | 8 |
| Soft tissue release | 9 |
| Orthoses, mobility aids and aids for daily living | 15 |
| Assistive technology | 13 |
| Multidisciplinary team | 13 |

Table S4: Curriculum content on neck and low back pain

| Topic | Number of schools |
| --- | --- |
| Mechanical | 20 |
| Inflammatory | 20 |
| Destructive | 19 |
| Crush fracture related | 18 |
| Red-flag signs/symptoms* | 19 |
| Spinal stenosis | 19 |
| Radiculopathy | 19 |
| Spondylolysis | 14 |
| Spondylolisthesis | 13 |

*should lead to investigation for serious pathology

Table S5: Curriculum content on fibromyalgia and regional pain syndromes

| Topic | Number of schools |
| --- | --- |
| Fibromyalgia |  |
| Symptoms | 19 |
| Signs | 19 |
| Criteria for diagnosis | 17 |
| Sleep abnormality | 17 |
| Abnormalities in pain physiology | 16 |
| Screening investigations for co-morbid conditions | 16 |
| Other functional pain syndromes and psychosocial distress | 16 |
| Regional pain/overuse |  |
| Presentations | 20 |
| Risk factors | 20 |
| Outcomes | 20 |

Table S6: Curriculum content on bone joint and soft-tissue malignancy

| Topic | Number of schools |
| --- | --- |
| Bone metastases (symptoms, signs, differential diagnosis, investigations) | 15 |
| Myeloma (symptoms, signs, differential diagnosis, investigations) | 12 |
| Primary tumours of bone and soft-tissues* | 12 |
| Pigmented villonodular synovitis | 2 |
| Hypertrophic pulmonary osteo-arthropathy | 2 |
| Paraneoplastic rheumatological presentations | 1 |

*A description of clinical presentation in 12, outline of the classification and morphology in 10, and pathological consequences in 11 curricula.

Table S7: Curriculum content on uncommon RMDs

| Topic | Number of schools |
| --- | --- |
| Overlap syndromes including mixed connective tissue diseases | 2 |
| Anti-synthetase syndrome | 1 |
| Auto-inflammatory diseases | 1 |
| Ehlers Danlos syndrome | 2 |
| Arthropathy due to haemophilia | 2 |
| Osteogenesis imperfecta | 1 |
| Muscular dystrophy | 1 |
| Diabetic cheiro-arthropathy | 2 |


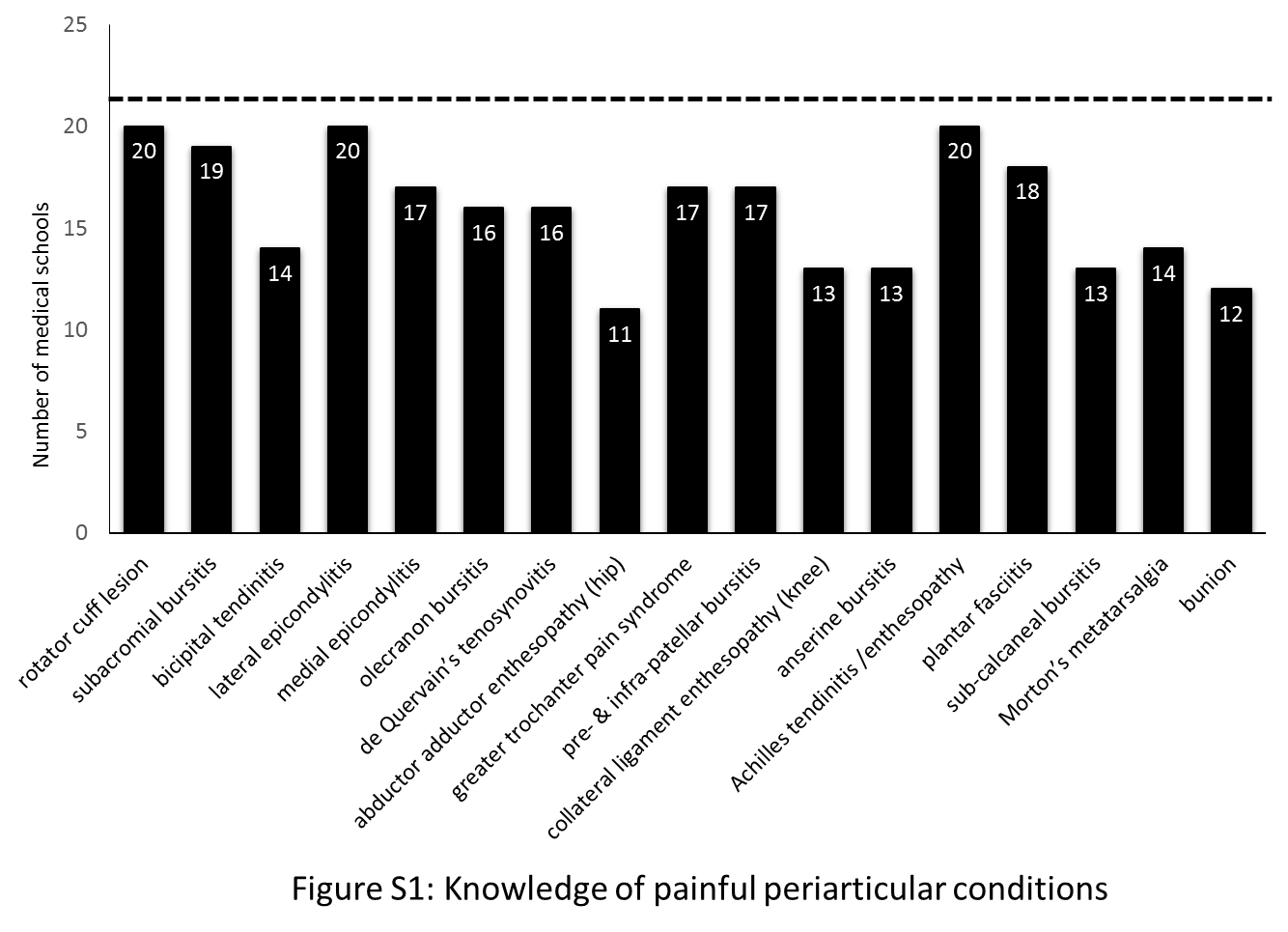


Figure S1: Curriculum content on painful peri-articular lesions


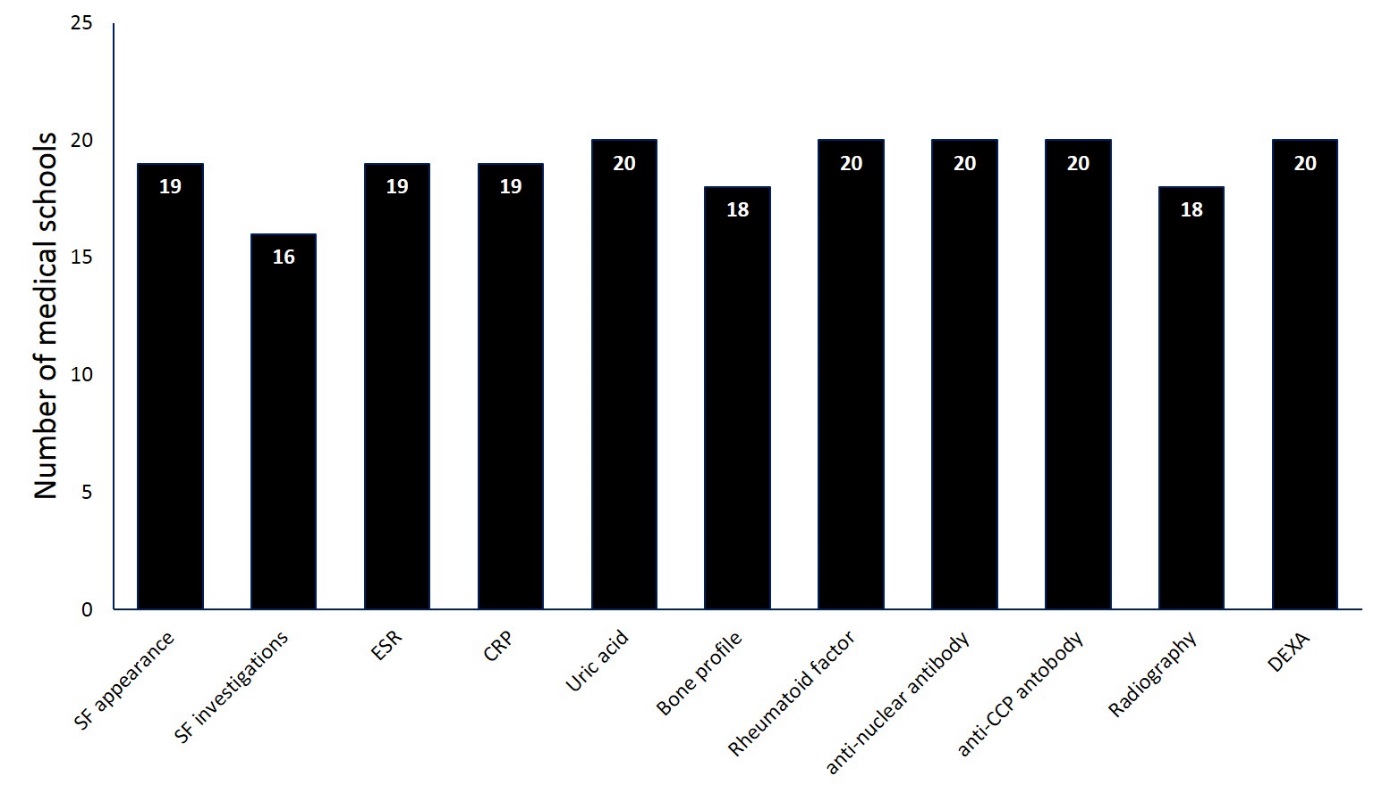
Figure S2: Curriculum content on investigations relevant to musculoskeletal diseases. Musculoskeletal, salivary and vascular ultrasonography; and Magnetic Resonance Imaging (MRI), Computerised Tomography (CT) were included in the curriculum of three, one, and one; and two and one schools, respectively.


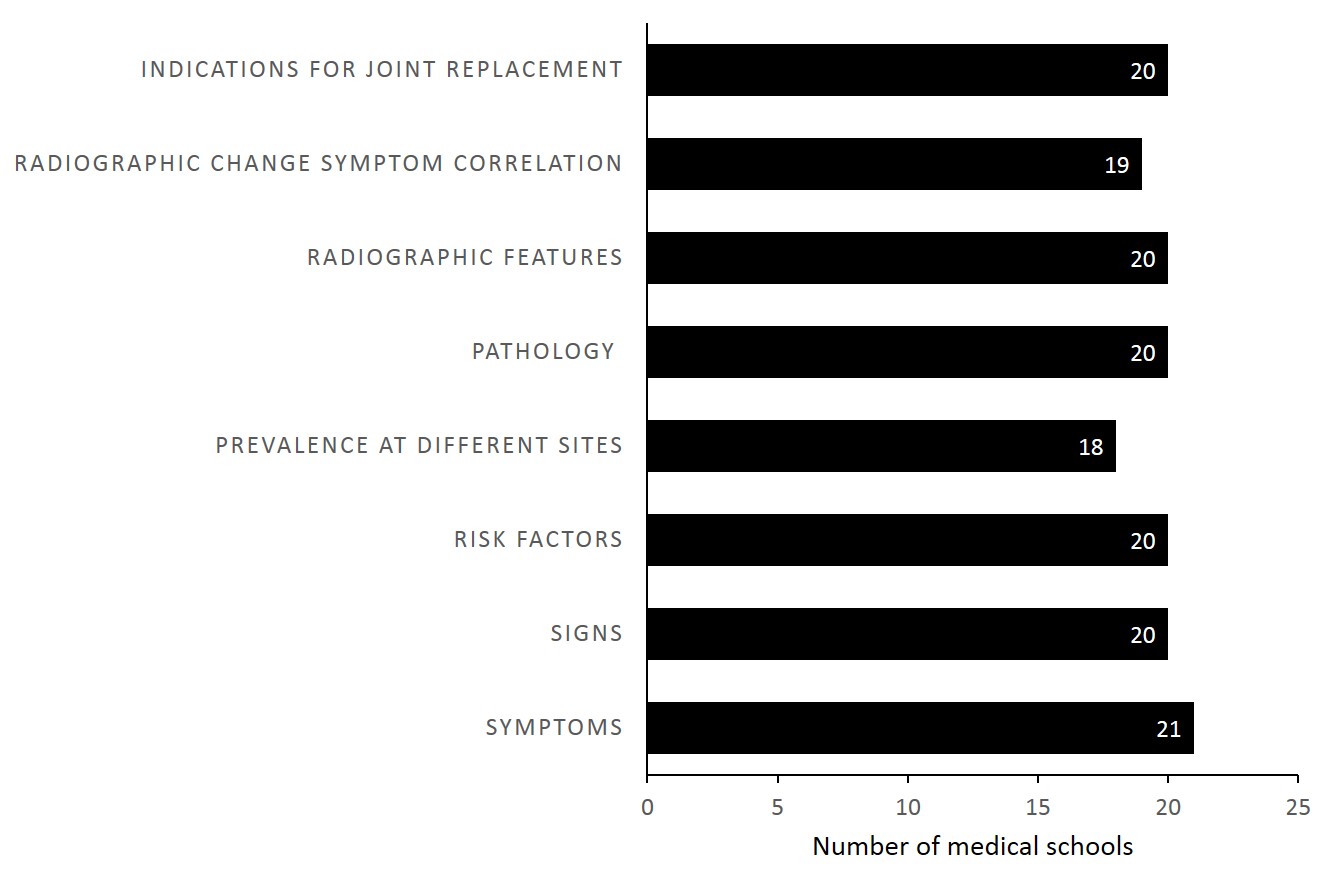
Figure S3: Curriculum content on osteoarthritis


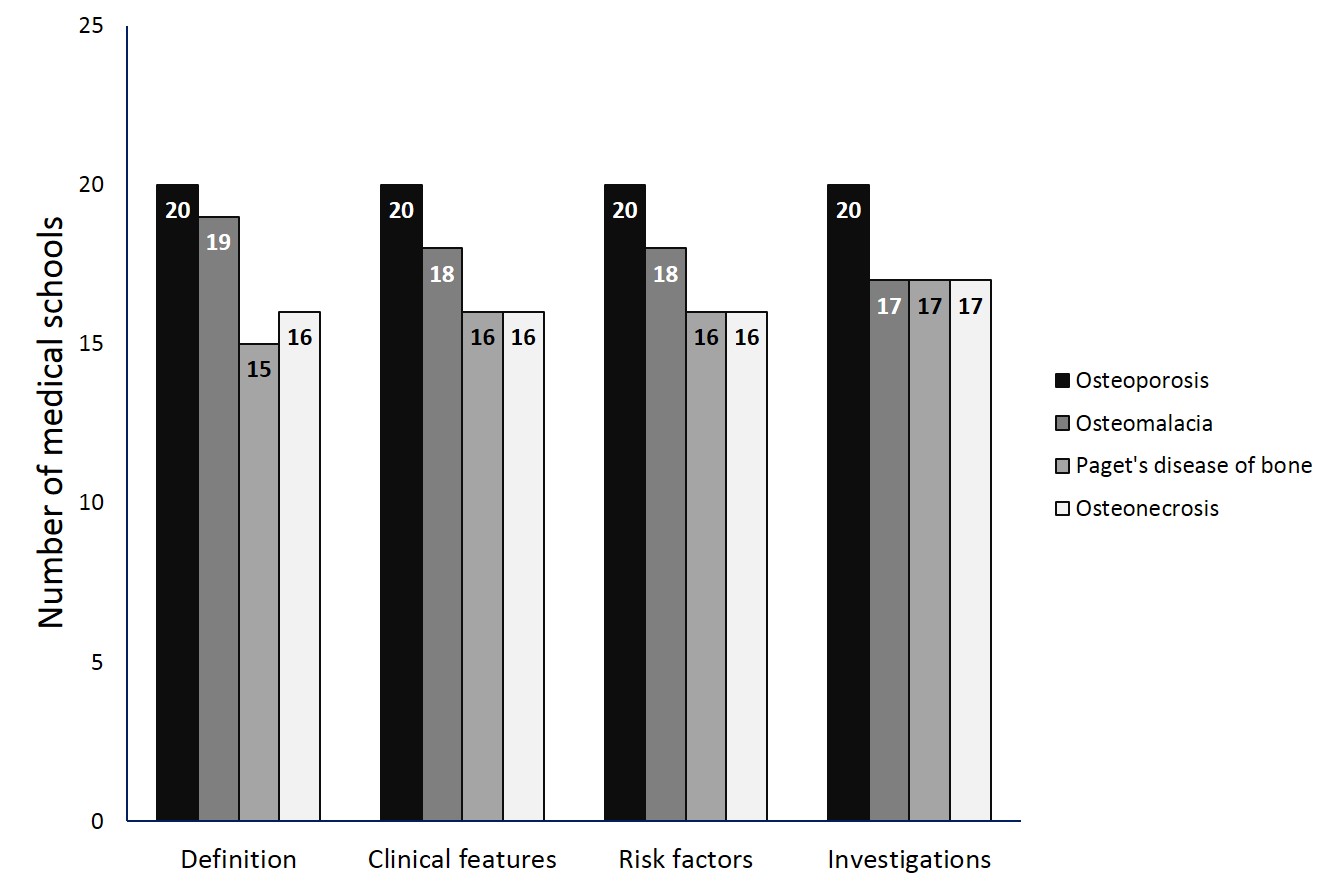


Figure S4: Curriculum content on metabolic bone diseases


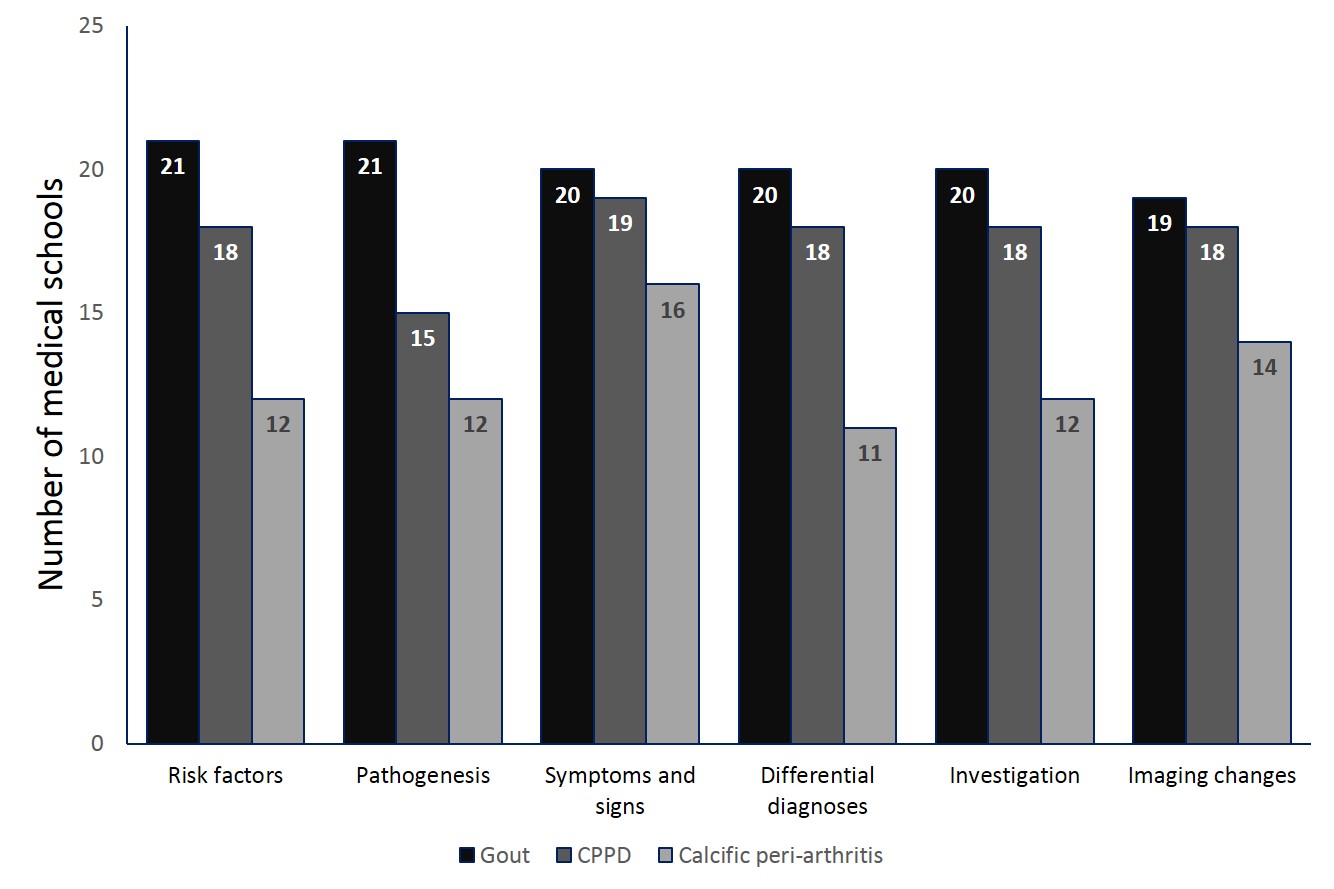


Figure S5: Curriculum content on microcrystalline arthropathies
